# A Genetic Screen for Olfactory Habituation Mutations in *Drosophila*: Analysis of Novel *Foraging* Alleles and an Underlying Neural Circuit

**DOI:** 10.1371/journal.pone.0051684

**Published:** 2012-12-17

**Authors:** Mark Eddison, Amsale T. Belay, Marla B. Sokolowski, Ulrike Heberlein

**Affiliations:** 1 Department of Anatomy, University of California San Francisco, San Francisco, California, United States of America; 2 Department of Ecology and Evolutionary Biology, University of Toronto, Toronto Ontario, Canada; Alexander Flemming Biomedical Sciences Research Center, Greece

## Abstract

Habituation is a form of non-associative learning that enables animals to reduce their reaction to repeated harmless stimuli. When exposed to ethanol vapor, *Drosophila* show an olfactory-mediated startle response characterized by a transient increase in locomotor activity. Upon repeated exposures, this olfactory startle attenuates with the characteristics of habituation. Here we describe the results of a genetic screen to identify olfactory startle habituation (OSH) mutants. One mutation is a transcript specific allele of *foraging* (*for*) encoding a cGMP-dependent kinase. We show this allele of *for* reduces expression of a *for-T1* isoform expressed in the head and functions normally to inhibit OSH. We localize *for-T1* function to a limited set of neurons that include olfactory receptor neurons (ORNs) and the mushroom body (MB). Overexpression of *for-T1* in ORNs inhibits OSH, an effect also seen upon synaptic silencing of the ORNs; *for-T1* may therefore function in ORNs to decrease synaptic release upon repeated exposure to ethanol vapor. Overall, this work contributes to our understanding of the genes and neurons underlying olfactory habituation in *Drosophila*.

## Introduction

Habituation is a fundamental behavior that is often overlooked by researchers, yet its prevalence in the animal kingdom suggests it is essential for survival [1]. Habituation is an active process of progressive decline of reaction to a harmless stimulus [2], [3]. Habituation allows animals to ignore inconsequential stimuli and may serve as a building block for more complex forms of attention [4]. An inability to habituate has been linked to schizophrenia [5], [6], autism [7], [8] and fetal alcohol syndrome [9], [10]. Despite the biological and clinical importance of habituation, its behavioral simplicity and its first description over 100 years ago [11], few genes that govern habituation have been described to date.

Our understanding of the neural basis of habituation is most extensive in the sea snail *Aplysia californica* [12], [13], whose defensive gill-withdrawal reflex habituates to repeated mechanical stimulation [14]–[16]. Early work showed that habituation in this sensory-neuron to motor-neuron circuit is due to a presynaptic decrease in excitatory neurotransmission, likely due to the active silencing of presynaptic release [12]. Although this decrease in presynaptic release, termed homosynaptic depression, is a common mechanism of habituation, potentiation of inhibitory connections can also achieve the same behavioral output [17], [18].

A variety of paradigms have been used to study habituation in *Drosophila* including the gustatory-based proboscis extension reflex (PER) and several olfactory-mediated behaviors, such as the jump reflex or startle response [19]. Using reverse genetics, several well studied genes and pathways have been identified as important regulators of habituation in *Drosophila*. These include K^+^ channels, NMDA and GABA_A_ receptors, as well as the cAMP and cGMP second messenger systems [18], [19]. An unbiased forward genetic approach can be useful in identifying novel genes and pathways that regulate habituation. However, due to the labor-intensive nature of many habituation assays, this approach has only sparsely been used [20], [21].

Our laboratory has previously described a simple and efficient paradigm to study olfactory startle habituation (OSH) in freely moving adult *Drosophila* [22]. In this assay, the flies’ gradual decline of a locomotor startle response to short exposures of vaporized ethanol is measured using an automated video tracking system [22]. The organization of the *Drosophila* olfactory system shows remarkable similarities to that of vertebrates [23], [24], suggesting the principal genes, circuits and mechanisms of olfactory habituation maybe conserved. In flies, odors are detected by the olfactory receptor neurons (ORNs), most of which reside in the antennae and project to the antennal lobe (AL) where they synapse with glomerulus-specific projection neurons (PNs) and local interneurons (LNs) [24]. Both excitatory and inhibitory LNs are present in the AL and make intra- and inter-glomerular connections with the PNs, shaping the neural representation of odors from the 1^st^ to 2^nd^ order neurons [25]–[27]. The PNs project to the mushroom body (MB) and lateral horn. Importantly, the MB is critical for habituation [22], [28], associative olfactory learning [29], and modulating locomotor responses [30]. Recently, much progress has been made in deciphering the neural circuits and identifying several genes mediating olfactory habituation in *Drosophila* [17], [18], [20], [31], [32].

In this report we describe the results of a genetic screen using our OSH paradigm [22] and identify 26 mutations affecting OSH. We also further characterize two hypomorphic mutations in the gene *foraging (for)*, which encodes a cGMP-dependent kinase (PKG). These new *for* mutations decrease the expression of a specific isoform of *for*, *for-T1*. We show that *for-T1* normally functions to inhibit OSH in a subset of neurons that include ORNs and the MB. We also show that overexpression of *for-T1* in ORNs, but not the MB, reduces OSH, suggesting that *for-T1* principally functions in ORNs to regulate OSH. Finally, we show that synaptic transmission of ORNs is required to promote startle habituation. Taken together, our results raise the possibility that *for-T1* may inhibit OSH by decreasing synaptic release in ORNs after their exposure to ethanol vapor.

## Materials and Methods

### Fly Strains

All flies were maintained on standard cornmeal molasses agar at 25°C and 70% humidity under constant dim light. The P element collection screened was a collective effort generated internally in the Heberlein Lab. *NP2614* was obtained from GETDB (*Drosophila* Genetic Resource Centre in Kyoto Institute of Technology). Our two control strains, 4.59 and 16.57 have P elements inserted just 5′ of *CG5630* and in *Socs36E* respectively, genes with no known association with habitation or PKG. *UAS-TeTx* and *UAS-TeTx^in^* strains were obtained from Sean Sweeney, *pdf-GAL4* flies were obtained from Paul Tagert, and *Orco-GAL4* from Leslie Vosshall. *UAS-for-T1* and *for^R^, for^s^* and *for^s2^* were obtained from Marla Sokolowski. *OK-107-GAL4*, *UAS-GFP-CD8* and the septate junction P elements were from the Bloomington Stock Centre. All strains, except the *for* polymorphisms, were backcrossed for at least five generations to a *w^1118^ Berlin* stock.

### Habituation Assay

The habituation assay is essentially the same as described in [22], except that we used the “booz-o-mat” [66] which allows simultaneous video recording of eight individual genotypes. Films were recorded using Adobe Premiere (Adobe Systems, San Jose, CA). To measure the locomotor tracking response to ethanol, films were analyzed with a modified version of DIAS 3.2 (Solltech, Oakdale, IA) that was controlled by the OneClick 2.0 scripting language (Westcode Software, San Diego, CA). Briefly, for each genotype, 20 2-4 day-old male flies were collected under CO_2_ anesthesia and kept in fresh food vials for 2 days. Flies were placed into a 16×125 mm cylindrical tube with perforations clustered at the rounded base. Flies were left to acclimate for 7 min before the start of video recording. After a further two minutes the flies were administered the first 30-second pulse of vaporized ethanol (P1); subsequent 30-second pulses of ethanol vapor were administered every 5 minutes. One minute after the forth pulse (P4) the flies were dishabituated with a sudden mechanical shock (banging the apparatus). A final pulse of ethanol vapor was administered 4 minutes after the dishabituation. Ethanol vapor was produced with an evaporator [22], [66] and the concentration controlled by a flow meter (Cole Parmer). Mixtures of ethanol and air vapor are noted as ratios. For screening purposes, a ratio of ethanol/air of 65/77 was used. All subsequent testing was carried out at an ethanol/air ratio of 80/60, where 80 units of flux is equivalent to 2.7 liters/min. Habituation assays were repeated on 2 to 3 different days with new flies to incorporate the day-to-day variations in behavior. In all Figures, *n* corresponds to the number of experiments performed on an independent group of 20 flies.

### Calculations and Statistics

The total movement travelled during odor exposures was calculated as the area under the pulse curve, i.e. summing the velocities measured during the 30-second exposure, at 5-second intervals and multiplying the sum by 5 seconds. The habituation index (HI) was calculated as 1-P4/P1, where P4 and P1 are the areas under the locomotor activity curve for the 4^th^ and 1^st^ pulse respectively, such that a HI of 1 indicates complete habituation and a HI of 0 indicates no habituation. In order to more easily compare the extent of habituation in all graphs the total movement was normalized to the magnitude of the first startle. Significance was established by one-way-ANOVA with post-hoc Newmans-Keuls comparisons. Error bars in all experiments represent standard error of the mean (SEM). Statistical significance was achieved where p<0.05.

### Genetic Screen

A total of 874 P-element insertion strains were initially screened in the habituation assay, (n = 2–4). The habituation index (HI) for each strain was calculated and ranged from 0.92 to –0.42. A frequency distribution of the habituation indices showed a near normal distribution with a mean of 0.58, median of 0.64 and mode of 0.65. From 874 strains screened, 93 were identified, 63 had pronounced habituation (with an HI >0.8) and 30 failed to habituate (with an HI <0.2). These strains were backcrossed for 5 generations to our *w^1118^ Berlin* genetic background to eliminate unlinked mutations. After retesting in the habituation assay (n = 6), 26 strains maintained their habituation phenotype: 25 exhibited enhanced habituation (HI>0.8) and 1 was a non-habituator (HI<0.2). All mutant strains were considered to be within the normal range of a locomotor startle response as none were signficantly different than at least one of the control strains (see below). Further, all of these mutant strains appeared healthy, fertile and viable. Two representative backcrossed strains, 4.59 and 16.57, that had a normal HI similar to the screen median and mode (0.49 and 0.5, respectively) and initial startle (36.7 mm/fly and 29.5 mm/fly, respectively) were chosen as controls and used throughout the behavioral experiments, although only one control strain is shown.

### Molecular Characterization of for Alleles

The location of the insertions was determined by inverse PCR. (The *11.247* P element is located in the first intron of *for*, 994 bp downstream of exon 1, and the *NP2614* insertion is located in the same intron, 666 bp downstream of exon 1. Imprecise excision strains of *11.247* were generated through remobilization of the P[GawB] element by introduction of a stable transposase source. Several phenotypic revertants were obtained. The excision strains were screened by PCR on genomic DNA using primers 5′-ACTACGCTACGCTGGCAGAAAC-3′ and 5′-AACACGAACACGA AAGATTGG -3′, and several were found to be precise excisions.

### RNA Analysis

Total RNA was extracted from adult flies using Trizol Reagent (Invitrogen). Poly A^+^ RNA was purified from total RNA using the Oligotex system (Qiagen). Probes for the Northern blot were generated by PCR using genomic DNA as a template. For the *for-T1* probe, the primers used were 5′-ATCTGGTGGGTGGCATTGTGA-3′ and 5′-CATCCTTGTCGTATTTGGGAAA-3′. For the *for-T2*, the primers were 5′-AGGAACACGAACTGGAAG-3′ and 5′-GATACAGAAACCCTCCCCGTTA-3′. As a control for RNA loading, a *tubulin 84B* gene probe was amplified using primers 5′-ACAGCCGTCTCTAGCTCCG-3′ and 5′-CATCACCTCCGCCCACGGTCTTG-3′. Northern blots were performed using mRNA isolated from 2–4 day old adult heads and bodies (or heads only) and probed with ^32^P labeled probes.

### PKG Enzymatic Activity Assay and Immunohistochemistry

PKG enzyme assays and immunohistochemistry were performed as previously described [44].

## Results

### A Behavioral Screen for Mutations Affecting Olfactory Startle Habituation

**Figure 1:**
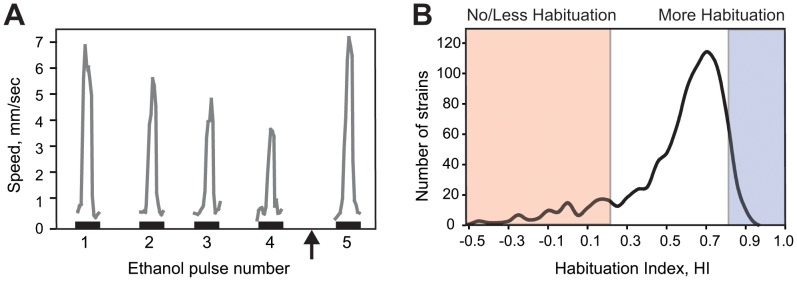
*Drosophila* habituates to an ethanol-induced olfactory startle. **A)** The olfactory startle attenuates with the characteristics of habituation. Ethanol naïve flies exposed to a 30-sec pulse of ethanol vapor showed an olfactory mediated startle response, characterized by a transient increase in locomotor activity. In subsequent pulses of ethanol vapor the olfactory startle increasingly attenuated. To demonstrate habituation (and not sensory adaptation or fatigue) flies were dishabituated (arrow) with a mechanical stimulus before the final pulse of ethanol. **B)** The frequency distribution of habituation indices (HI) of all strains tested in the genetic screen. The habituation index was calculated as a ratio of the total movement in the fourth pulse (P4) and first pulse (P1) (HI = 1-P4/P1; a HI of 1 indicates complete habituation while a HI of 0 or lower indicate no habituation or sensitization, respectively). A frequency distribution of the habituation indices showed a near normal distribution with a mean of 0.58, median of 0.64 and mode of 0.65. A HI of 0.8 or higher was used as the cut-off for enhanced habituation, while a HI of 0.2 or lower was the cut-off for failure to habituate normally. Strains with low and high HIs were selected for further analysis.

In *Drosophila*, exposure to a high concentration of ethanol vapor provokes a transient olfaction-dependent increase in locomotor activity termed the olfactory startle response [22]. Subsequent pulses of ethanol vapor result in a reduced startle that shows characteristics of habituation, including dishabituation following a novel stimulus (Fig. 1A; [22]). In order to identify genes that regulate OSH, we screened 874 fly strains, each harboring a randomly inserted P element in the genome, for strains with altered OSH. As a simple measure for habituation we calculated a habituation index (HI), defined as the ratio between the magnitudes of the fourth and first startle response (see Methods). We defined a normal HI to be similar to the median and mode of the entire screen and selected two strains as controls (termed *Ctrl*, see Methods). Potential mutants were selected by a numerical cut-off point at both ends of the distribution of HIs obtained from the screen (see Fig. 1B; see Methods).

After eliminating unlinked mutations by backcrossing each potential mutant to the parental strain (*wBerlin*; see Methods), we identified 26 strains that had a normal initial startle response and retained an abnormal OSH (Table 1). Curiously, only one of these strains decreased OSH (strain 12.132, which essentially failed to habituate), while all other strains had enhanced OSH. Using inverse PCR and DNA sequencing followed by genomic database searches (www.flybase.org) we mapped the location of the transposon insertions and identified the candidate genes disrupted (Table 1). Classified by their molecular function, the largest categories were those including genes with predicted or unknown molecular function (*lama*, *ckn, hebe, CG1806, CG8321, CG3967, CG42697, CG11357*) and genes with functions related to nucleic acids (*HmgD, tara, Camta*, *heph*, *snp*). Smaller categories included genes involved in cell signaling (*for, gish, wun, PNUTS*), the regulation of the cytoskeleton (*kl-2, RtnL1)* or cell junctions (*pyd*, *cora*).

**Table 1:** OSH mutants isolated from genetic screen.

Strain	Initial Startle (mm/fly)	Habituation Index	P-element orientation and candidate gene affected	Molecular class	Nucleotide Insertion
**9.181**	35.0	0.85	**⇒** in ***caskin***	CGd	10850830
**9.189**	26.6	0.82	**⇐** in ***hephaestus***	RB	27811472
**9.197**	32.8	0.83	**⇒** in ***gilgamesh***	CS	12106609
**10.66**	41.5	0.86	**⇐** in ***High mobility group protein D***	DB	17601579
**11.158**	36.3	0.83	**⇐** 5′ of ***snap***	RB	17948455
**11.244**	23.3	0.83	**⇐** in ***taranis***	DB	12056400
**11.247**	34.1	0.84	**⇒** in ***foraging***	CS	3655713
**11.272**	24.0	0.80	**⇒** in ***polychaetoid***	CA	4720698
**12.112**	31.5	0.82	**⇒** in ***wunen***	CS	5297595
**12.132**	31.6	0.05	**⇐** in ***Calmodulin-binding transcription factor***	DB	5339712
**12.19**	31.3	0.84	**⇒** 5′ of ***PNUTS***	CS	870364
**12.82**	31.5	0.83	**⇒** 5′ ***CG1806***	CG	11901097
**12.95**	37.5	0.83	**⇒** 5′ of ***ade5***	M	12654602
**12.167**	38.1	0.86	**⇐** 5′ of ***coracle***	CA	15116495
**12.171**	40.0	0.85	**⇒** in ***CG8321***	CG	7922098
**12.172**	39.3	0.83	**⇒** in ***Rtnl1***	CY	5001033
**12.222**	34.5	0.86	**⇒** in ***CG3967,*** 5′ of ***astray***	CG, CS	9416260
**14.29**	32.6	0.82	**⇒** in ***male fertility factor kl2***	CY	132704
**14.86**	27.1	0.83	**⇐** in ***CG42697***	CG	14499267
**18.56**	36.6	0.87	**⇒** in ***Puromycin sensitive aminopeptidase***	PP	1517272
**18.94**	27.0	0.82	**⇒** 5′ of ***Peroxiredoxin 2540-1***	CGd	6310865
**18.104**	31.7	0.86	**⇐** in ***hephaestus***	RB	27811472
**19.28**	30.2	0.82	**⇒** in ***starvin***	PP	13473388
**19.47**	26.4	0.85	**⇐** in ***CG11357***	CGd	4542807
**19.70**	40.9	0.85	**⇒** in ***lamina ancestor***	CG	5348461
**21.28**	40.2	0.85	**⇒** 5′ of ***hebe***	CG	5724097

Initial startle: distance moved per fly during first 30-second startle. Arrows represent direction of the P element. Molecular classes: cell signalling (CS), DNA binding (DB), RNA binding (RB), cell adhesion (CA), cytoskeleton (CY), metabolism (M), proteases (PP), annotated genes unknown molecular function without homology (CG), and those annotated genes with conserved structural domains (CGd). Information current to FlyBase release: FB2012_05, Sept 7th, 2012.

### Several Olfactory Startle Habituation Mutations are Associated with Septate Junctions

One candidate gene we identified in our screen, *coracle (cora)*, encodes an integral component of septate junctions [33]. In the insect nervous system, septate junctions are known to seal neighboring glial cells together to protect axons from the high K^+^ environment of the hemolymph [34]. Septate junctions are found in the fly’s blood-brain barrier, between perineurial and peripheral glia, and also between peripheral glia and axons [35]. An analogous structure in mammals is the paranodal junction found at the nodes of Ranvier, which enables rapid saltatory conduction of action potentials [36]–[38]. Interestingly, a parallel screen for OSH mutations identified *gliotactin* (*gli*) (B. Cho and U.H, unpublished data), another component of the septate junction [39], [40]. The identification of two septate junction genes in our screens suggested that the structure and/or function of the septate junction might be important for OSH. To test this hypothesis, we selected fifteen P element mutations in eight known genes whose products localize to the septate junctions, normalized their genetic background and tested their OSH response. Of these, seven strains, representing five of the eight genes tested (*cora*, *dlg*, *fas3*, *gli*, *nrx-IV*), showed an abnormal OSH (Table S1). Further, one strain (EP809, inserted in *nrx-IV*) failed to habituate, a phenotype rarely seen in the original screen. Therefore, septate junctions maybe an important regulator OSH, though future work will be necessary to reveal its specific function in modulating this form of behavioral plasticity.

### Foraging Regulates Olfactory Startle Habituation

**Figure 2:**
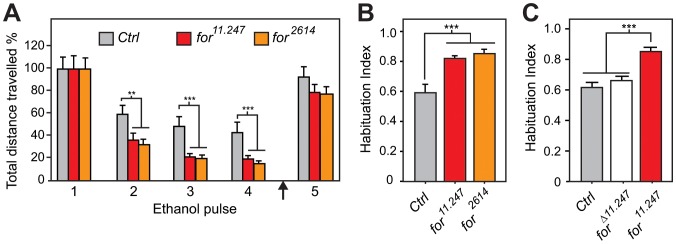
*for* alleles enhance olfactory startle habituation. **A)** *for^11.247^* and *for^2614^* show enhanced OSH. A reduction of distance traveled (compared to *Ctrl*) was seen in both alleles at pulse 2 (p<0.01), 3 and 4 (p<0.001;(n = 12). **B)** *for^11.247^* and *for^2614^* have an enhanced HI (indicating more habituation). Significant difference was seen between *Ctrl* and *for^11.247^* or *for^2614^* (p<0.001; n = 12). **C)** Compared to *Ctrl,* the precise excision, *for^Δ11.247^,* had a normal HI (p>0.05; n = 6). Unless indicated, significance was established by a One-Way-ANOVA with post-hoc Newmans-Keuls tests in all figures (*p<0.05, **p<0.01, ***p<0.001).

**Figure 3:**
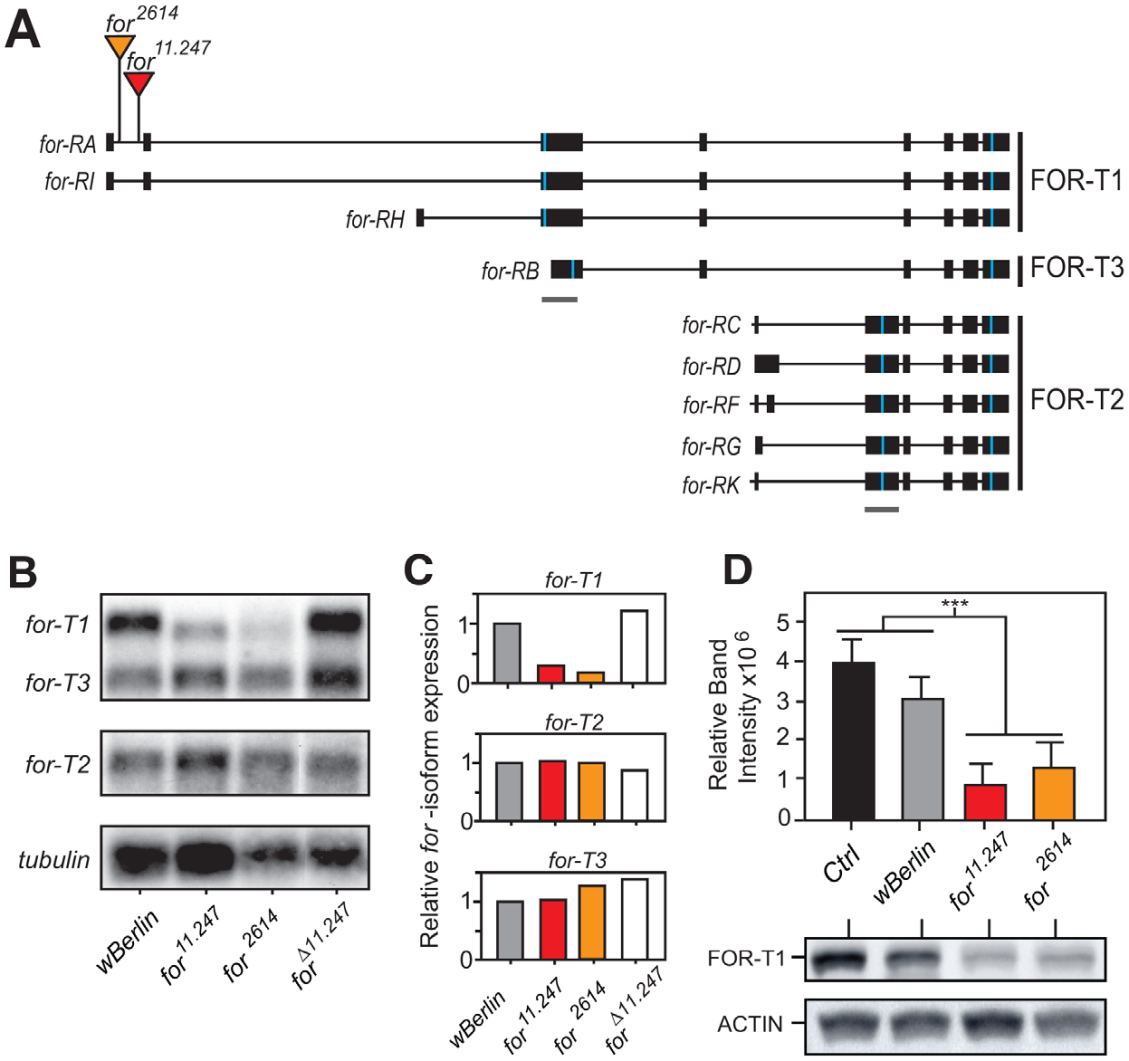
Molecular characterization of *for* alleles. **A)** Schematic of the *for* transcription unit, with insertion sites of *for^11.247^* and *for^2614^*. Blue bars represent translation start/stop sites, grey bars represent region probed for *for-T1/T3* and *for-T2* transcripts. The 3 major *for* isoforms, collectively called *for-T1/T2/T3* have a total of nine splice forms, all encoding a common kinase domain at the 3′ end. FOR-T1 is a 1088 amino acid (aa) protein encoded by *for-RA/RH*/*RI*, FOR-T2 is a 894 aa protein encoded by *for-RC/RD/RF/RG/RK*, and FOR-T3 is a 742 aa protein encoded by *for-RB*. **B)** Northern blot of adult fly mRNA using probes specific to *for-T1/T3* or *for-T2* transcripts. **B, top panel)** In the control strain (*wBerlin*) we detected two bands with the *for-T1/T3* probe. Based on its size, the upper, more intense, band corresponds to *for-T1* transcripts, while the lower, less intense band, to the *for-T3* transcript. Compared to *wBerlin* and *for^Δ11.247^*(a precise excision of *for^11.247^*) a reduced intensity of *for-T1*, but not *for-T3* transcripts, was seen in *for^11.247^* and *for^2614^*. **B, middle panel)** Using a *for-T2* probe we detected no differences in levels of *for-T2* transcripts in either *for^11.247^* or *for^2614^*. **B, bottom panel)** A *tubulin* probe was used to compare total mRNA levels. **C)** Quantification of Northern Blot showing reduced *for-T1,* but not *for-T2* or *for-T3,* in *for^11.247^* and *for^2614^*. Levels were calculated as a ratio between *for* and *tubulin* band intensity. **D)** Quantification and representative Western blot of extracts from adult heads analyzed with an antibody that recognizes FOR-T1. Compared to controls, we saw a reduction of FOR-T1 in both *for^11.247^* and *for^2614^* (p<0.001; n = 3).

In *Drosophila*, *foraging* (*for/dg2*) encodes a cGMP-dependent kinase (PKG) that regulates habituation of the giant-fiber neurons (involved in an escape reflex) [41] and the PER [42]. We identified strain 11.247, carrying a P element insertion in the 5′ region of the *for* locus (from hereon called *for^11.247^*), that exhibited enhanced habituation (Fig. 2A, 2B). This phenotype is robust as it was maintained in two different genetic backgrounds (Fig. S1A). Moreover, normal habituation was restored upon precise excision of the P element in *for^11.247^* (Fig. 2C), indicating that the insertion is responsible for the mutant phenotype. We also identified an additional strain that carries a P element insertion near *for^11.247^*, NP2614 (called *for^2614^*; see Fig. 3A), which also enhanced OSH (Fig. 2A, 2B). In both of these *for* alleles, the magnitude of the initial startle was normal (Fig. S1B). Therefore, we conclude that *for* regulates OSH.

### Molecular Characterization of for Alleles that Disrupt Olfactory Startle Habituation

The *for* locus produces 11 transcripts that encode four protein isoforms [43]. Of these transcripts, nine encode the three major FOR protein isoforms FOR-T1, T2 and T3 (Fig. 3A; see [43] for alternative nomenclature). To determine the molecular nature of our *for* alleles, we performed Northern blots using mRNA derived from adult flies. In control flies we observed two bands using probes specific for the *for-T1* and *for-T3* transcripts (Fig. 3B, top panel). The more intense, larger molecular weight band corresponds to the three *for-T1* transcripts, *for-RA/RI/RH*, while the lower molecular weight less intense band corresponds to *for-T3* (or *for-RB*). With a probe specific to *for-T2* transcripts we detected a doublet corresponding to *for-RD/RF* and *for-RC/RG/RK* (Fig. 3B; middle panel).

In both *for* alleles we observed a reduced intensity of the *for-T1* band and did not detect a change in either the *for-T3* or *for-T2* transcripts (Fig. 3B, 3C). Therefore, both *for^11.247^* and *for^2614^* have specifically reduced expression of *for-T1* transcripts. The precise excision of the P element in *for^11.247^* that showed normal habituation (Fig. 2C) also restored *for-T1* transcripts to control levels (Fig. 3B, 3C, top panel). Therefore, our data suggest that *for-T1* functions to inhibit OSH.

Using a FOR antibody [44], we next determined levels of FOR-T1 and FOR-T3 in the adult heads of flies carrying these *for* alleles; we were unable to determine FOR-T2 levels with this antibody. We observed a significant reduction in FOR-T1 protein expression in *for^11.247^* and *for^2614^* (Fig. 3C), while levels of FOR-T3 appeared normal (Fig. S1C). Therefore, consistent with the Northern blot, we conclude that, both *for^11.247^* and *for^2614^* have reduced expression of FOR-T1 in the adult head.

We next attempted to measure the level of PKG activity in the heads of *for^11.247^* and *for^2614^* flies. However, the levels of PKG activity in our P element control strain and genetic background control were significantly different (Fig. S2A), precluding any definitive conclusions about relative levels of PKG activity in *for^11.247^* and *for^2614^* (which were also significantly different from each other). We also failed to see a difference in OSH in the natural variants of *for* (*for^R^, for^s^* and *for^s2^*) that do subtly but significantly differ in PKG activity [44], [45] (Fig. S2B). Therefore, for reasons we do not currently understand, we were unable to find a correlation between PKG activity, levels of *for-T1* and OSH. In summary, we conclude that *for^11.247^* and *for^2614^* have reduced levels of *for-T1* in the adult head suggesting that *for-T1* functions to inhibit OSH.

### for^11.247^-GAL4 Expression Partially Recapitulates the FOR Expression Pattern

**Figure 4:**
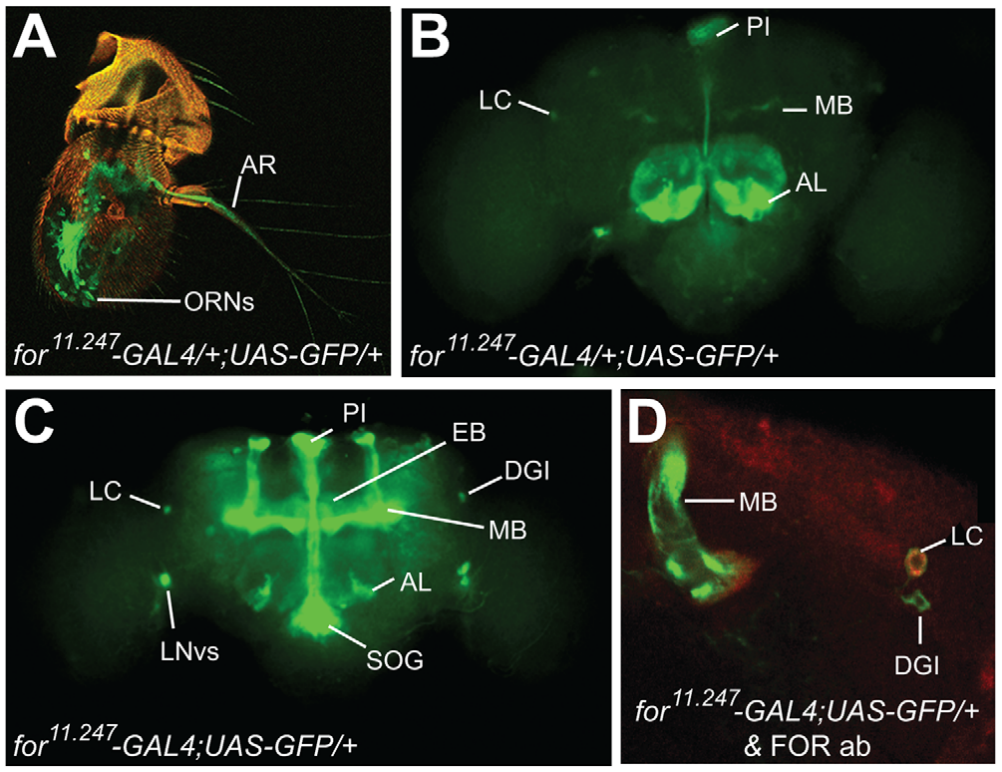
Expression pattern of *for^11.247^-GAL4*. **A, B)** Expression of *for^11.247^-GAL4/+;UAS-GFP/+* flies. **A)** In the antenna, GFP (green) was expressed in the arista (AR) and a sub-population of olfactory receptor neurons (ORNs) in the third antennal segment. **B)** In the CNS, GFP was expressed in specific glomeruli in the antennal lobe (AL), par intercrebalis (PI) neurons, and weakly in the mushroom body (MB) and lateral cells (LC). **C)** In *for^11.247^-GAL4;UAS-GFP/+* flies strong GFP expression was seen in MB, PIs, LC and sub-oesophageal ganglion (SOG), as well as the ventral lateral neurons (LN_v_s), the giant dorsal interneuron (DGI), parts of the antennal lobe (AL) and ellipsoid body (EB). **D)** Higher magnification of *for^11.247^-GAL4;UAS-GFP/+* flies showing partial co-localization with a FOR antibody (red) in the MB and LC, but not in DGI.

The neuronal expression pattern of all FOR isoforms has been reported previously and includes specific neuroanatomical loci, namely the ellipsoid body (EB), mushroom body (MB), dorsal posterior cells (DPC) and clusters of neurons situated laterally [44], [46]. Since the P elements in the *for* alleles drive GAL4 expression [47], its insertion in/near the 5′ end of *for-T1* may capture the endogenous *for-T1* expression pattern. Expression of *GFP* with *for^11.24^* ^7^ *-GAL4* or *for^2614^-GAL4* (in flies of genotype *for-GAL4/+;UAS-GFP/+*) revealed expression in the aristae (AR), a subset of ORNs of the 3^rd^ antennal segment, discrete glomeruli of the antennal lobe (AL), the pars intercerebralis (PIs), and very weakly in the MB and lateral cell (LC) (Fig. 4A, 4B, data not shown). In *for^11.247^* homozygotes (flies of genotype *for-GAL4;UAS-GFP/+*) we observed stronger GFP expression in the MB, as well as additional expression in the EB and the *pigment-dispersing factor* (PDF)-expressing ventral lateral neurons (LN_v_s) (Figs. 4C, S3A–C).

This GFP expression pattern partially overlaps, in the MB and LN, with that observed with the FOR antibody [44]. We did not, however, detect GFP expression in the DPCs, which stain with a FOR antibody (Fig. S3D); thus DPCs may not express the FOR-T1 isoform of FOR. Expression of GFP was also observed in regions not labeled by the FOR antibody, including the ORNs, LN_v_s and PI neurons. However, our behavioral data suggests that FOR is likely expressed in ORNs (see below). Furthermore, mammalian PKG is expressed in ORNs and the suprachiasmatic nucleus [48]. Therefore, FOR may not be expressed at levels detectable by this FOR antibody in the ORNs, LN_v_s and some neurons of the PI.

### for-T1 Functions in for^11.247^-GAL4 Neurons to Inhibit Olfactory Startle Habituation

**Figure 5:**
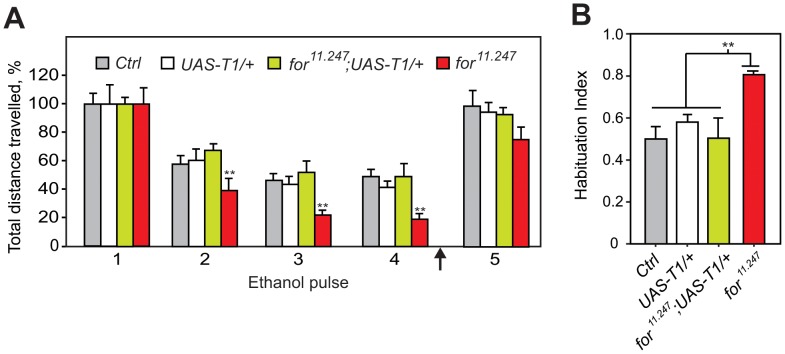
*for^11.247^ -GAL4* flies expressing *for-T1* have normal olfactory startle habituation. **A)** Habituation profile of functional rescue of *for^11.247^-GAL4* by expressing *UAS-for-T1*. No significant difference in distances travelled were seen between *for^11.247^-GAL4*;*UAS-for-T1/+* and either *Ctrl* or *UAS-for-T1/+.* At pulse 2, 3 and 4, a significant difference was only seen between *for^11.247^–GAL4*;*UAS-for-T1/+* and *for^11.247^* (p<0.01; n = 12). **B)** HI of *for* ^11.247^ rescue. No significant differences were seen between *for^11.247^-GAL4*;*UAS-for-T1/+* and either *Ctrl* or *UAS-for-T1/+*, but were observed between *for^11.247^-GAL4*;*UAS-for-T1/+* and *for^11.247^* (p<0.01; n = 12).

In order to test if *for-T1* functions in the neurons defined by *for^11.247^-GAL4*, we attempted to rescue the enhanced habituation of *for^11.247^* by expressing *for-T1* with *for^11.247^-GAL4*. Indeed, expressing *for-T1* in homozygous *for^11.247^-GAL4* flies restored normal OSH (Fig. 5A, 5B). We conclude that the enhanced OSH of *for^11.247^* flies is due to reduced *for-T1* expression and that *for-T1* function in *for^11.247^ -GAL4* neurons is sufficient for flies to show normal habituation.

### Synaptic Silencing of ORNs Inhibits Olfactory Startle Habituation

**Figure 6:**
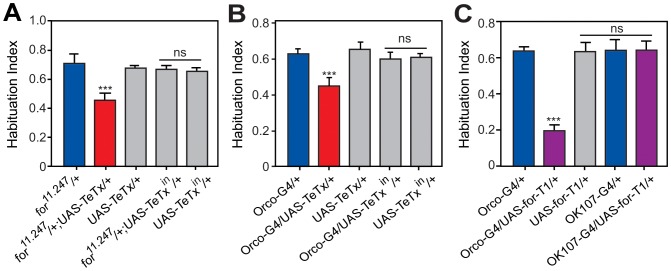
Analysis of neuronal circuitry implicated in olfactory startle habituation. **A)** Blocking synaptic activity in *for^11.247-GAL4^* neurons reduces OSH. Heterozygous *for^11.247^-GAL4* flies expressing tetanus toxin (*TeTx*) had reduced OSH. Significant differences were seen between *for^11.247^-GAL4* ***/*** *+* or *UAS-TeTx/+* and *for^11.247^-GAL4/+;UAS-TeTx/+* (p<0.001; n = 9). No significant difference was seen in flies expressing inactive *TeTx* (*TeTx^in^*) with *for^11.247^-GAL4* ***/*** *+* (p>0.05; n = 9). **B)** Synaptic silencing of ORNs inhibits OSH. Expressing *UAS-TeTx* with *Orco-GAL4* significantly reduced OSH. Differences were observed between *Orco-GAL4/+* or *UAS-TeTx/+* and *Orco-GAL4/+;UAS-TeTx/+* (p<0.01; n = 6). No effect was seen upon expressing *UAS-TeTx^in^* with *Orco-GAL4/+* (p>0.05; n = 9). **C)** *for-T1* overexpression in ORNs inhibits OSH. Expressing *UAS-for-T1* with *Orco-GAL4*, but not with MB driver *OK107-GAL4*, reduced OSH. Significant differences were observed between *Orco-GAL4/+* or *UAS-for-T1/+* and *Orco-GAL4/+*;*UAS-for-T1/+* (p<0.001; n = 14), but not between controls and *OK107-GAL4/+;UAS-for-T1/+* (p>0.05; n = 8).

We next investigated whether the activity of neurons expressing *for^11.247^-GAL4* directly regulates OSH. To test this, we first blocked synaptic release in *for^11.247^-GAL4/+* expressing neurons with tetanus toxin light chain (*TeTx*) [49]. Blocking synaptic release by expressing *TeTx* in *for^11.247^-GAL4/+* expressing neurons reduced OSH (Fig. 6A), without affecting the initial startle (Fig. S4A). Further, expressing an inactive form of *TetTx* (*TeTx-^in^*) in *for^11.247^-GAL4/+* expressing neurons did not alter OSH (Fig. 6A). These data suggest that synaptic activity of the ORNs, a few MB neurons and/or PI neurons promotes habituation. To further define the neurons regulating OSH, we next silenced specific subsets of *for^11.247^-GAL4/+* expressing neurons. As blocking synaptic release in the MB is already known to regulate OSH [22], we focused on other neurons of the *for^11.247^-GAL4*-expression pattern, specifically the ORNs and LN_v_s.

To test if the activity of the ORNs promotes habituation, we silenced them by expressing *TeTx* with the *Odorant receptor co-receptor*-GAL4 (*Orco-GAL4*) driver, which is expressed in ∼80% of ORNs [50]. Like *for^11.247^-GAL4/+* expressing neurons, synaptic silencing of the ORNs also significantly suppressed OSH (Fig. 6B), without affecting the initial startle (Fig. S4B). Therefore, neurotransmission in the ORNs defined by *Orco-GAL4* is required to promote OSH.

Since the PDF-expressing LN_v_s are also labeled by *for^11.247^–GAL4* (Fig. S3A-C), we next tested if synaptic activity of the LN_v_s neurons regulates OSH. However, silencing neurotransmission in the LN_v_s, by expressing *TeTx* with *Pdf-GAL4* [51], did not affect OSH (Fig. S4C). Therefore, our data suggest that LN_v_ neurons do not regulate OSH. In summary, our data indicate that synaptic activity of ORNs promotes OSH.

### FOR-T1 Overexpression in the ORNs Inhibits Olfactory Startle Habituation

We have shown that *for-T1* inhibits OSH (Fig. 5) and that blocking synaptic release in ORNs, which likely express *for-T1* (Fig. 4A), also reduces OSH (Fig. 6B). Therefore, it is possible that *for-T1* inhibits OSH by decreasing synaptic release in ORNs. If this was the case, increasing levels of *for-T1* in ORNs should reduce OSH. Indeed, similar to the effect of silencing ORNs with *TeTx*, overexpression of *for-T1* with *Orco-GAL4* significantly reduced OSH (Fig. 6C). Therefore, *for-T1* may inhibit OSH by reducing synaptic release in ORNs. We also tested whether *for-T1* overexpression in MB affects OSH. However, flies expressing *for-T1* with the pan-MB driver *OK107-GAL4* had a normal OSH (Fig. 6C), suggesting that in the MB *for-T1* may not regulate OSH. To conclude, our data suggest that *for-T1* may act primarily in ORNs to inhibit OSH and a possible FOR-T1 function here is reduction of synaptic release after an initial exposure to ethanol vapor.

## Discussion

We describe the isolation of *Drosophila* mutants that disrupt olfactory startle habituation (OSH); of these 26 mutants, the majority showed enhanced OSH. Additional targeted analysis also identified several strains carrying mutations in genes that play a role in septate junctions thus implicating this structure in regulating OSH. We characterized two mutations in *for* that enhanced OSH due to reduced expression of a specific *for* product, FOR-T1. We show that *for-T1* limits OSH by functioning in a subset of neurons that include ORNs and the MB. Our data further map *for-T1* function primarily to ORNs, implying that OSH can occur in the sensory neurons of the olfactory circuit.

*for* encodes several isoforms of protein kinase G (PKG), a cGMP-dependent serine/threonine kinase that regulates neuronal excitability [52] and leaning and memory [46]. With respect to habituation, the natural variant (*for^s^*) with reduced PKG activity [45] also has reduced habituation of the giant-fiber system, which mediates escape responses to visual stimuli [41] and the gustatory-based PER [42], implying that *for* limits these behaviors. We now show that *for* also limits OSH; thus *for* appears to be a central suppressor of habituation, regardless of sensory modality. A question remains as to whether *for* isoforms and their function is similar in these separate neuronal populations. Interestingly, the mammalian PKG with highest homology to *for*, PRKG1, [53] has been associated with Attention Deficit/Hyperactivity Disorder [54], a condition characterized by a persistent lack of attention possibly due to a failure to habituate to large amounts of information received from the environment [55].

Ethanol activates several olfactory receptors (ORs): OR7a, OR22a, OR35a, OR85b (http://neuro.uni-konstanz.de/DoOR). Although, curiously, activity of the ORNs expressing these ORs does not appear to be needed for flies to initially sense the smell of ethanol, as the magnitude of the initial startle response was unaffected by synaptic silencing using *Orco-GAL4*. Interestingly, one glomerulus that appeared labeled in *for^11.247^-GAL4* heterozygotes is VC31, which expresses OR35a, the OR most strongly activated by acute ethanol. Therefore, VC31 maybe a glomerulus mediating ethanol-induced OSH. It is also worth noting that, in addition to activating particular ORs, ethanol is also a known GABA_A_ receptor agonist [56] and may also act on GABA_A_ receptors expressed in LNs and PNs that promote OSH [18], [32], [57].

How might *for-T1* function in ORNs to limit OSH? Since *for-T1* overexpression in ORNs, or their synaptic silencing, reduced OSH, *for-T1* may limit OSH by decreasing synaptic release. Indeed, cultured neurons of *for^s^* flies with reduced PKG activity [45] exhibit increased excitability, resulting in increased spontaneous and evoked activity [52]. *for-T1* may achieve decreased synaptic release by modulating cAMP levels, as PKG does in mammalian ORNs [58], [59]. Alternatively, as in the mammalian neurons, it may phosphorylate a number of possible substrates including: TRPC channels, which regulate Ca^2+^ influx [60], SEPTIN3, a regulator of vesicle targeting or tethering [61], [62], or transporters of serotonin [63], [64], a neurotransmitter implicated in presynaptic inhibition in the AL [57].

Finally, our data suggest that olfactory habituation can occur in the 1^st^ order neurons of the olfactory circuit (the ORNs), while several recent papers demonstrate that the 2^nd^ order neurons of the olfactory circuit (the LNs and PNs) are key players in olfactory habituation [17], [18], [27], [32]. MB silencing and ablation experiments also suggest that these 3^rd^ order neurons are also involved [20], [22]. Indeed, studies in the rat show that olfactory cortex and not peripheral circuits, regulate olfactory habituation [65]. Therefore, the capacity to habituate to olfactory cues appears to be distributed throughout the olfactory circuit. Indeed, synaptic silencing of either the ORNs (this study) or the MB [22] did not completely block OSH, as one might expect if habituation occurred at a singular point in the circuit. This distributed mechanism of habituation may allow the fruit fly a greater flexibility in the interplay between its innate responses and learnt experience.

## Supporting Information

Figure S1 ***for^11.247^*** **has enhanced OSH in two genetic backgrounds. A)** *for^11.247^* in the *wBerlin* background has enhanced OSH (p>0.001; n = 7, Unpaired t-test). *for^11.247^* in the *2202U* isogenic background has enhanced OSH (p>0.0134; n = 7, Unpaired t-test). **B)** *for* alleles have a normal initial startle response. Total movement during the first ethanol pulse was similar between *Ctrl*, *for^11.247^* and *for^2614^* (p>0.05; n = 8). **C)** FOR-T3 are unaffected in *for^11.247^* and *for^2614^*. Representative Western blot of adult heads using an antibody that recognizes FOR-T3. Compared to controls**,** no differences in levels of FOR-T3 were observed.
(TIF)

Figure S2 **PKG activity levels do not correlate with OSH or** ***for-T1*** **levels. A)** Levels of PKG activity levels were significantly different between control strains *wBerlin* and *Ctrl* (p<0.001; n = 5), precluding informative conclusions about PKG activity in *for^11.247^* and *for^2614^*, which were also significantly different from each other (p<0.001; n = 5). **B)** *for^R^*, *for^s^* and *for^s2^* did not show significant differences in OSH (p>0.05; n = 6–8).
(TIF)

Figure S3 ***for^11.247^-GAL4*** **is expressed in PDF expressing neurons, but not DPC neurons. A)** Expression of *GFP* (green) in *for^11.247^-GAL4* flies revealed expression in the lateral ventral neurons (LN_v_s), identified in **(B)** by a PDF antibody (red). **C)** Co-localization of GFP and PDF in *for^11.247^–GAL4;UAS-GFP/+* flies. **D)** Co-staining of *for^11.247^-GAL4;UAS-GFP/+* flies with FOR antibody (red), revealed no co-localization in the dorsal posterior cells (DPCs).
(TIF)

Figure S4 ***for^11.247^-GAL4*** **and** ***Orco-GAL4*** **neurons expressing TeTx have a normal initial startle. A)** No difference in total movement in the initial startle was seen between *for^11.247^-GAL4/+;UAS-TeTx/+, for^11.247^-GAL4/+* and *UAS-TeTx/+* (p>0.05; n = 9). **B)** No difference in total movement of the initial startle was seen between *Orco-GAL4/+;UAS-TeTx/+, Orco-GAL4/+* and *UAS-TeTx/+* (p>0.05; n = 6). **C)** Expressing Tetanus Toxin in PDF neurons did not alter OSH. No significant difference in HI was seen between *Pdf-GAL4/+;UAS-TeTx/+* and *Pdf-GAL4/+* or *UAS-TeTx/+* (p>0.05; n = 8–12).
(TIF)

Table S1 **Habituation Index of P elements inserted in or 5′ to septate junction genes.**
(DOCX)
