# Supplementary figures and images for: A Genetic Screen for Olfactory Habituation Mutations in Drosophila: Analysis of Novel Foraging Alleles and an Underlying Neural Circuit
Source: PLoS One. 2012 Dec 17;7(12):e51684. doi: 10.1371/journal.pone.0051684 (PMC3524188; doi:10.1371/journal.pone.0051684)

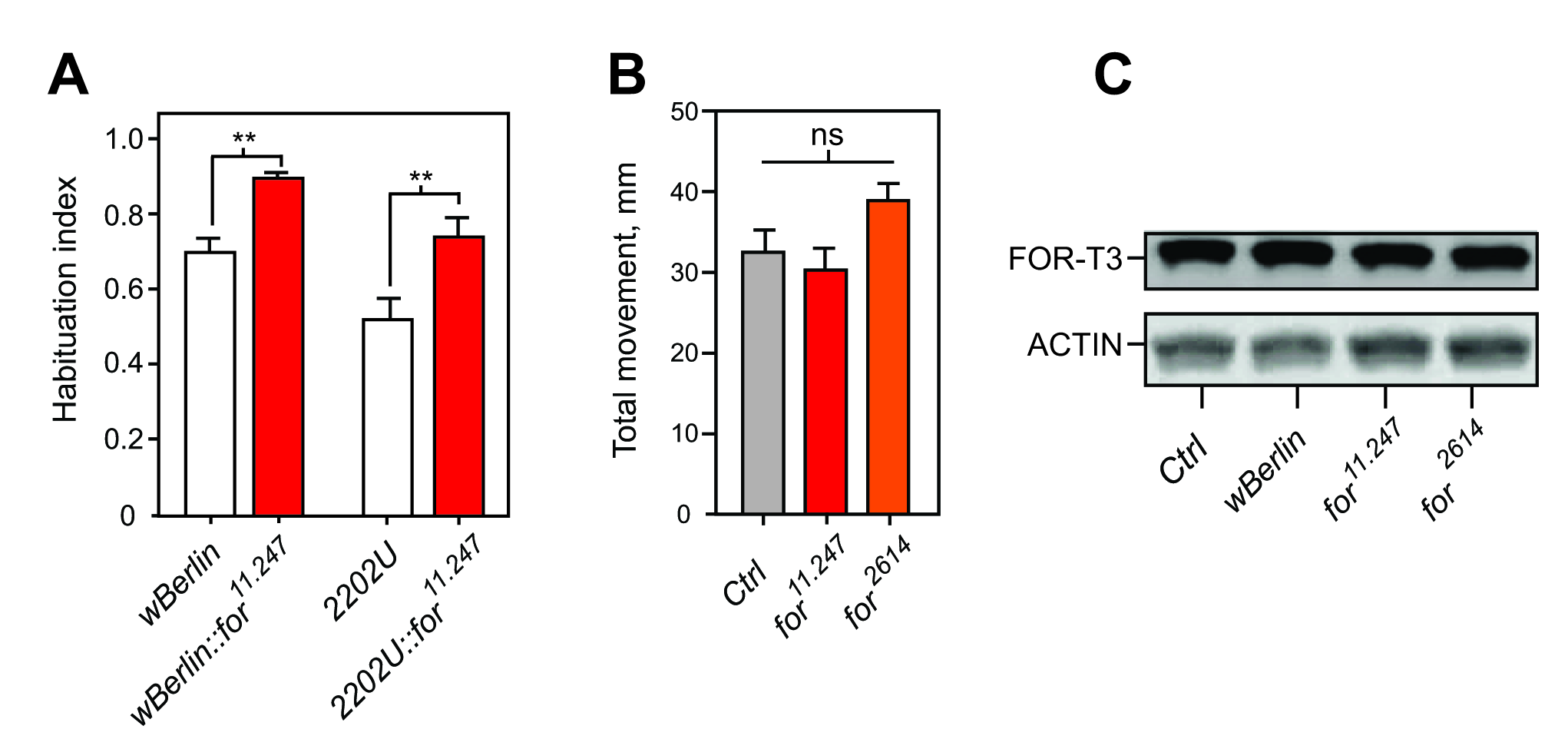

Supplement: Figure S1 — for11.247 has enhanced OSH in two genetic backgrounds. A) for11.247 in the wBerlin background has enhanced OSH (p>0.001; n = 7, Unpaired t-test). for11.247 in the 2202U isogenic background has enhanced OSH (p>0.0134; n = 7, Unpaired t-test). B) for alleles have a normal initial startle response. Total movement during the first ethanol pulse was similar between Ctrl, for11.247 and for2614 (p>0.05; n = 8). C) FOR-T3 are unaffected in for11.247 and for2614. Representative Western blot of adult heads using an antibody that recognizes FOR-T3. Compared to controls, no differences in levels of FOR-T3 were observed. (TIF) [file pone.0051684.s001.tif]

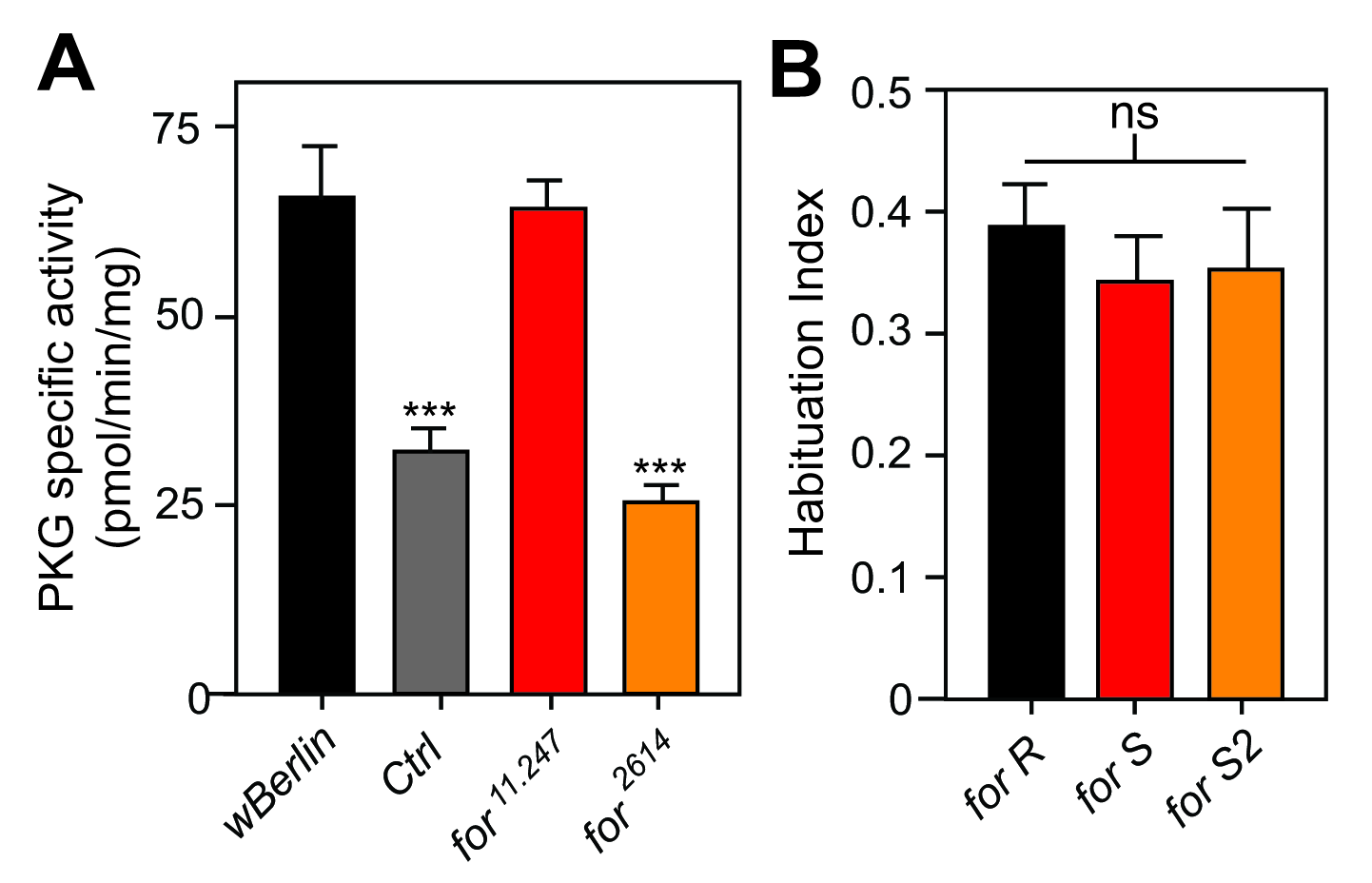

Supplement: Figure S2 — PKG activity levels do not correlate with OSH or for-T1 levels. A) Levels of PKG activity levels were significantly different between control strains wBerlin and Ctrl (p<0.001; n = 5), precluding informative conclusions about PKG activity in for11.247 and for2614, which were also significantly different from each other (p<0.001; n = 5). B) forR, fors and fors2 did not show significant differences in OSH (p>0.05; n = 6–8). (TIF) [file pone.0051684.s002.tif]

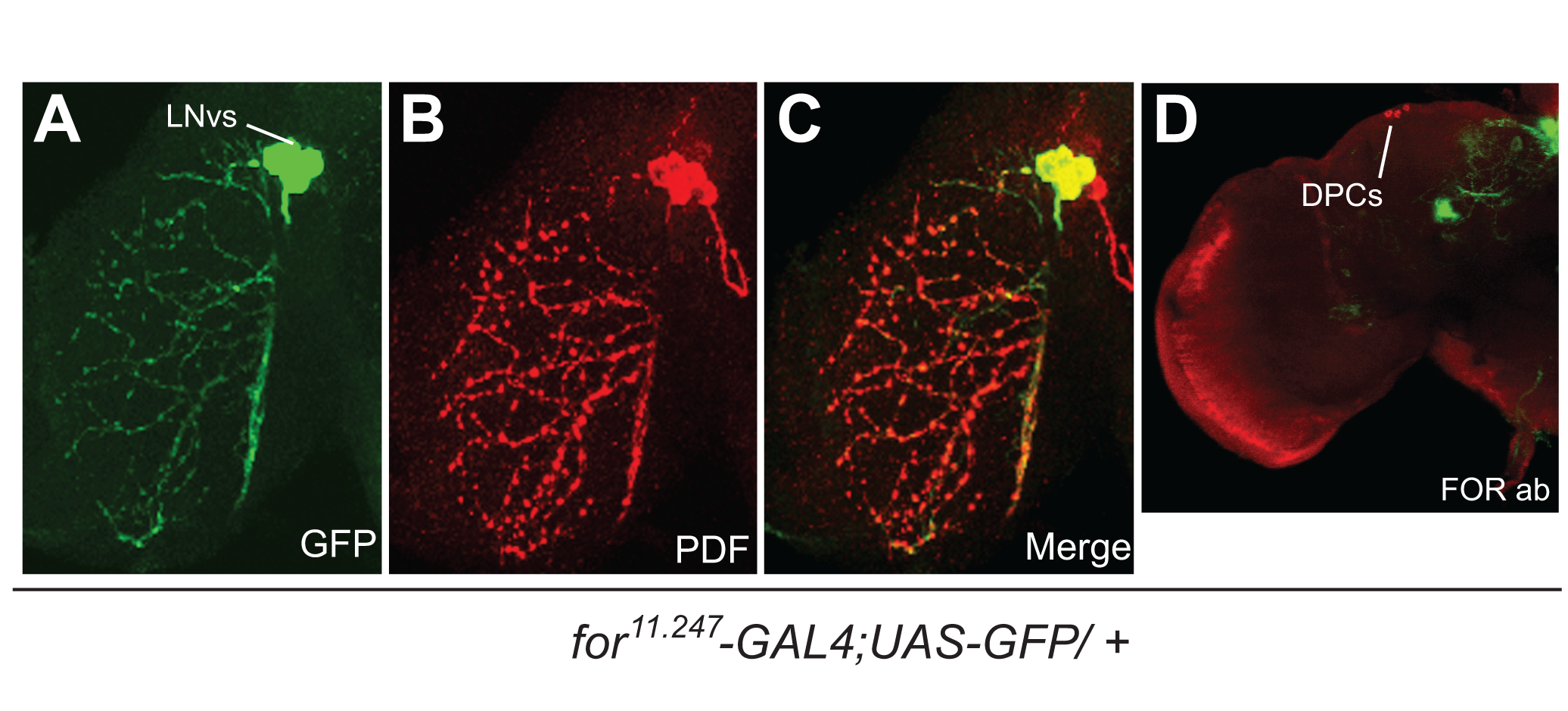

Supplement: Figure S3 — for11.247-GAL4 is expressed in PDF expressing neurons, but not DPC neurons. A) Expression of GFP (green) in for11.247-GAL4 flies revealed expression in the lateral ventral neurons (LNvs), identified in (B) by a PDF antibody (red). C) Co-localization of GFP and PDF in for11.247–GAL4;UAS-GFP/+ flies. D) Co-staining of for11.247-GAL4;UAS-GFP/+ flies with FOR antibody (red), revealed no co-localization in the dorsal posterior cells (DPCs). (TIF) [file pone.0051684.s003.tif]

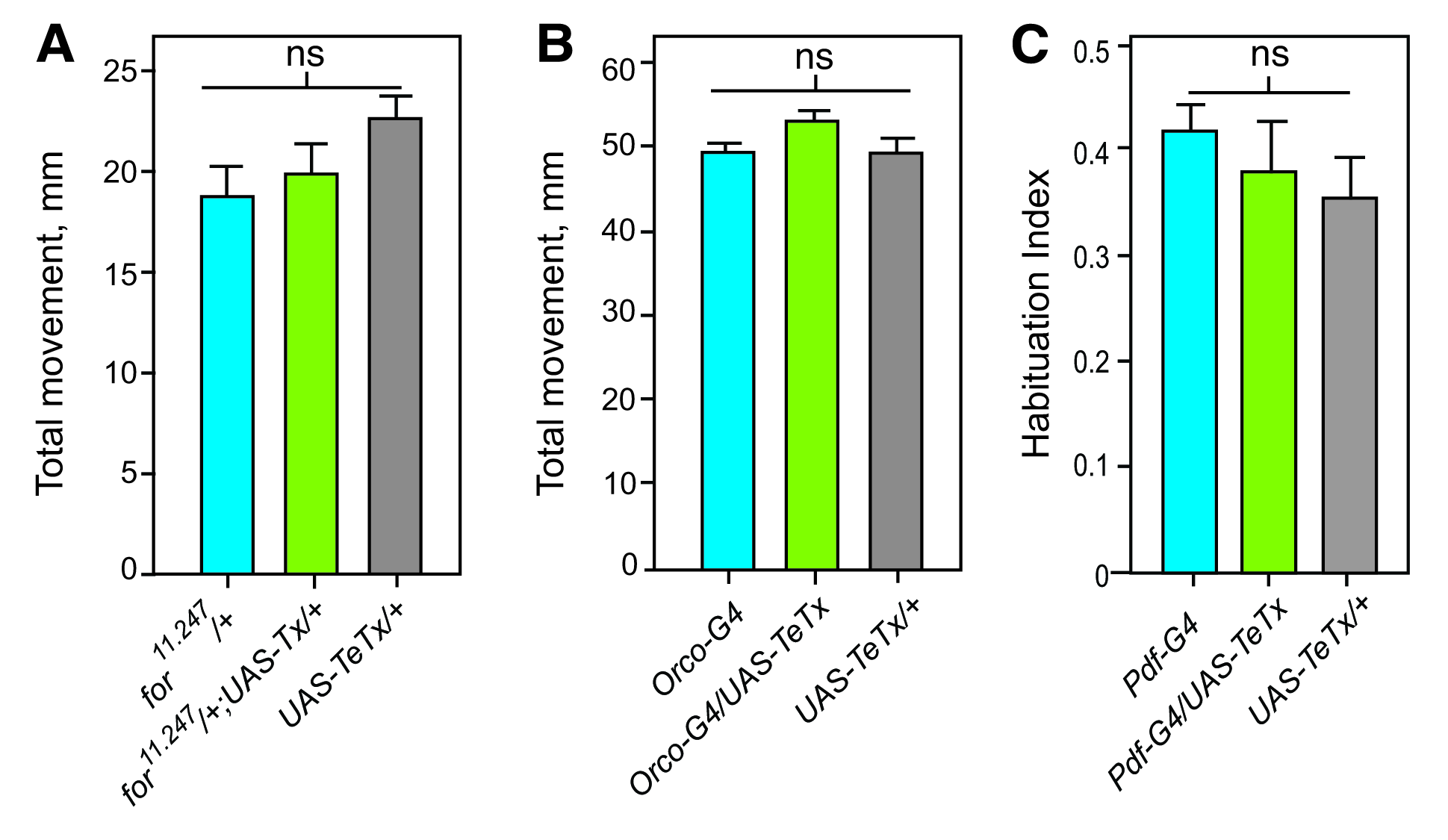

Supplement: Figure S4 — for11.247-GAL4 and Orco-GAL4 neurons expressing TeTx have a normal initial startle. A) No difference in total movement in the initial startle was seen between for11.247-GAL4/+;UAS-TeTx/+, for11.247-GAL4/+ and UAS-TeTx/+ (p>0.05; n = 9). B) No difference in total movement of the initial startle was seen between Orco-GAL4/+;UAS-TeTx/+, Orco-GAL4/+ and UAS-TeTx/+ (p>0.05; n = 6). C) Expressing Tetanus Toxin in PDF neurons did not alter OSH. No significant difference in HI was seen between Pdf-GAL4/+;UAS-TeTx/+ and Pdf-GAL4/+ or UAS-TeTx/+ (p>0.05; n = 8–12). (TIF) [file pone.0051684.s004.tif]
