# Supplementary material for: A Genetic Screen for Olfactory Habituation Mutations in Drosophila: Analysis of Novel Foraging Alleles and an Underlying Neural Circuit
Source: PLoS One. 2012 Dec 17;7(12):e51684. doi: 10.1371/journal.pone.0051684 (PMC3524188; doi:10.1371/journal.pone.0051684)
Supplement: Table S1 — Habituation Index of P elements inserted in or 5′ to septate junction genes. (DOCX) [file pone.0051684.s005.docx]

| \| **Candidate Gene** \| \| --- \| | \| **P element insertion** \| \| --- \| | \| **Habituation**  **Index** \| \| --- \| |
| --- | --- | --- | --- | --- | --- |
| \| *Atp-αlpha* \| \| --- \| | \| NP7509 \| \| --- \| | \| 0.65 \| \| --- \| |
| \| *coracle* \| \| --- \| | \| NP4290 \| \| --- \| | \| 0.80* \| \| --- \| |
|  | \| EY07598 \| \| --- \| | \| 0.57 \| \| --- \| |
| \| *discs-large* \| \| --- \| | \| NP4134 \| \| --- \| | \| 0.82* \| \| --- \| |
|  | \| NP7225 \| \| --- \| | \| 0.82* \| \| --- \| |
|  | \| NP768 \| \| --- \| | \| 0.73 \| \| --- \| |
|  | \| NP1102 \| \| --- \| | \| 0.78 \| \| --- \| |
| \| *fasciculin 3* \| \| --- \| | \| NP3393 \| \| --- \| | \| 0.79* \| \| --- \| |
|  | \| NP1233 \| \| --- \| | \| 0.76 \| \| --- \| |
| \| *gliotactin* \| \| --- \| | \| NP7092 \| \| --- \| | \| 0.82* \| \| --- \| |
|  | \| NP1106 \| \| --- \| | \| 0.85* \| \| --- \| |
| \| *neuroglian* \| \| --- \| | \| G00305 \| \| --- \| | \| 0.34 \| \| --- \| |
| \| *neurexin-IV* \| \| --- \| | \| EP809 \| \| --- \| | \| 0.18* \| \| --- \| |
| \| *scribbled* \| \| --- \| | \| EY04509 \| \| --- \| | \| 0.56 \| \| --- \| |
|  | \| NP7428 \| \| --- \| | \| 0.73 \| \| --- \| |

**Table S1:** **Habituation Index of P elements inserted in or 5’ to septate junction genes.** *designates a habituation phenotype using original screen criteria where >0.8 designates enhanced habituation and <0.2 designates a failure to habituate. For more information on P elements see flybase.org. All lines were backcrossed to the same genetic background (*wBerlin*).
